# Advanced age promotes colonic dysfunction and gut‐derived lung infection after stroke

**DOI:** 10.1111/acel.12980

**Published:** 2019-06-14

**Authors:** Shu Wen Wen, Raymond Shim, Luke Ho, Brooke J. Wanrooy, Yogitha N. Srikhanta, Kathryn Prame Kumar, Alyce J. Nicholls, SJ. Shen, Tara Sepehrizadeh, Michael de Veer, Velandai K. Srikanth, Henry Ma, Thanh G. Phan, Dena Lyras, Connie H. Y. Wong

**Affiliations:** ^1^ Department of Medicine, Centre for Inflammatory Diseases, School of Clinical Sciences Monash University Clayton Victoria Australia; ^2^ Department of Medicine (Academic Unit), Peninsula Clinical School, Central Clinical School Monash University Frankston Victoria Australia; ^3^ Department of Microbiology, Monash Biomedicine Discovery Institute Monash University Clayton Victoria Australia; ^4^ Monash Biomedical Imaging Monash University Clayton Victoria Australia; ^5^ Stroke and Ageing Research Group, Department of Medicine, School of Clinical Sciences, Monash Medical Centre Monash University Clayton Victoria Australia

**Keywords:** aging, bacteria, colon, infection, stroke

## Abstract

Bacterial infection a leading cause of death among patients with stroke, with elderly patients often presenting with more debilitating outcomes. The findings from our retrospective study, supported by previous clinical reports, showed that increasing age is an early predictor for developing fatal infectious complications after stroke. However, exactly how and why older individuals are more susceptible to infection after stroke remains unclear. Using a mouse model of transient ischaemic stroke, we demonstrate that older mice (>12 months) present with greater spontaneous bacterial lung infections compared to their younger counterparts (7–10 weeks) after stroke. Importantly, we provide evidence that older poststroke mice exhibited elevated intestinal inflammation and disruption in gut barriers critical in maintaining colonic integrity following stroke, including reduced expression of mucin and tight junction proteins. In addition, our data support the notion that the localized pro‐inflammatory microenvironment driven by increased tumour necrosis factor‐α production in the colon of older mice facilitates the translocation and dissemination of orally inoculated bacteria to the lung following stroke onset. Therefore, findings of this study demonstrate that exacerbated dysfunction of the intestinal barrier in advanced age promotes translocation of gut‐derived bacteria and contributes to the increased risk to poststroke bacterial infection.

## 1 INTRODUCTION

Stroke is a leading cause of death worldwide, contributing to over 10% of all deaths annually, and this is expected to rise due to an aging population. The incidence of stroke increases dramatically with age, with an estimated 70%–80% of all ischaemic strokes occurring in people above the age of 65 (Ovbiagele & Nguyen‐Huynh, 2011). Studies have highlighted the significance of age in worsening stroke severity, functional improvement and overall outcomes (Manwani et al., 2011; Ritzel et al., 2018; Yager, Wright, Armstrong, Jahraus, & Saucier, 2006). Specifically, the adverse biological effects of normal aging, such as cell senescence, low‐grade systemic inflammation and decline in immune function, can impend on events critical to recovery after stroke and contribute to the poor prognosis in the elderly (Licastro et al., 2005; Ritzel et al., 2018; Wen & Wong, 2018).

Despite its well‐recognized primary effects on the brain, a major cause of death after stroke is infection: a poststroke complication that has received increasing attention for its large clinical implications (Shim & Wong, 2018). In fact, more than 30% of infected patients with stroke die of infection as a secondary complication within a week of stroke onset, with infection of the respiratory (bacterial pneumonia) and urinary tracts most prevalent (Meisel, Schwab, Prass, Meisel, & Dirnagl, 2005). Advanced age and stroke severity are known early predictors for poor patient outcome after stroke (Wartenberg et al., 2011), possibly due to elevated risk of infection, but the underlying mechanisms are unclear.

Randomized clinical trials evaluating prophylactic antibiotics in patients with acute stroke showed that this therapeutic approach did not lower the incidence of poststroke pneumonia (Kalra et al., 2015), and it was not associated with reduced mortality or improved functional outcomes (Xi et al., 2017). These trials suggest a clear need for alternative treatment approaches, and importantly, a better understanding of the underlying mechanisms of poststroke infections. Associative clinical studies speculate that risk factors for poststroke pneumonia include dysphasia, nasogastric tubing, catheters, mechanical ventilation and aspiration (Chapman, Morgan, Cadilhac, Purvis, & Andrew, 2018). In addition, the age‐related decline in immune functions may further increase susceptibility of the elderly to poststroke infection (Crapser et al., 2016; Ritzel et al., 2018; Shaw, Goldstein, & Montgomery, 2013). While these factors may play an important role, our recent work demonstrated that poststroke infection may also originate from dissemination of gut commensal bacteria to peripheral tissues (Stanley et al., 2016). Indeed, >70% of the microorganisms detected in infected stroke patients of the studied cohort were common commensal bacteria that reside in the human intestinal tracts (e.g., *Enterococcus* spp., *Escherichia coli* and* Morganella morganii*) (Stanley et al., 2016). Unfortunately, a major caveat to these findings that demonstrate a vital brain‐gut link in the setting of stroke is that the effect of age was not assessed and warrants further investigation. As such, in this study, we examined if advanced age can promote the translocation and dissemination of commensal bacteria to potentiate the development of poststroke infection.

## 2 RESULTS

### 2.1 Advanced age is a risk factor for the development of infection in patients after acute stroke

A total of 633 patient records were obtained from the Monash Medical Centre, where 124 did not meet the inclusion criteria and were hence excluded, leaving a total of 509 within the study cohort. Patient cohort characteristics are summarized in Table 1. The mean patient age was 71.4 years (standard deviation [*SD*]: 14.5) with almost half being female (*n* = 226, 44.4%). A majority of patients presented with ischaemic strokes (*n* = 432, 84.9%) compared to haemorrhagic strokes (*n* = 77, 15.1%), but patients of the two stroke subtypes were similar with respect to age and sex distributions. A total of 55 (10.8%) patients from the cohort presented with a definite new case of infection after stroke as defined by the CDC/NHSH criteria. The most common types of infections were urinary tract infections (*n* = 24, 44.4% of all infections) and pneumonia (*n* = 21, 38.9%). Patients with multiple infections (*n* = 4, 7.4%) and other infections (eye/ear/nose/throat, gastrointestinal, other respiratory infections excluding pneumonia, skin/soft tissue, and unknown) were much less prevalent (*n* = 1, 1.8%).

**Table 1:** Patient cohort characteristics

	Patients (*n* = 509)
Age (mean/*SD*)	71.4 (*SD*: 14.5)
Female sex	226 (44.4%)
Days in acute ward (median)	4.5
Type of stroke at presentation	
Ischaemic	432 (84.9%)
Haemorrhagic	77 (15.1%)
Severity/NIHSS (mean)	9.84
Death	67 (13.2%)
Prevalence of infection	55 (10.8%)
Day of onset of infection (mean)	3.63
Site of infection (% of infected)	
Eye/ENT	1 (1.9%)
Gastrointestinal	1 (1.9%)
Lung (except pneumonia)	2 (3.7%)
Pneumonia	21 (38.9%)
Skin/Soft tissue	1 (1.9%)
Urinary tract	24 (44.4%)
Unknown	1 (1.9%)
Multiple	4 (7.4%)
Antibiotics prescribed	66 (13.0%)
IV line inserted	501 (98.4%)
IDC inserted	73 (14.3%)
NGT inserted	37 (7.3%)
ETT inserted	23 (4.5%)
Co‐morbid conditions	
Diabetes	122 (24.0%)
Chronic lung disease	49 (9.6%)
Chronic liver disease	6 (1.2%)
Malignancy	63 (12.4%)
HIV	0 (0.0%)
Autoimmune disease	13 (2.6%)
Medications	
Beta‐blockers	156 (30.6%)
Immunosuppressant	36 (7.1%)
Antibiotics (prestroke)	17 (3.3%)

Abbreviations: ENT, ear, nose and throat; ETT, endotracheal tube; HIV, human immunodeficiency virus; IDC, indwelling urethral catheter; IV, intravenous; NGT, nasogastric tube; *SD*, standard deviation.

Multivariable logistic regression modelling was used to determine the association between stroke and the presence of infection after adjusting for confounding factors: age, sex, stroke severity (NIHSS), immune‐suppressants, the presence of co‐morbidities, the use of indwelling urinary catheter (IDC) and the presence of nasogastric tube (NGT) feeding (Table 2). Multivariable regression model of risk factors analysis showed that age (OR 1.04, 95% 1.01–1.07), stroke severity (OR 1.05, 95% 1.01–1.08) and the use of an indwelling catheter (OR 7.79, 95% 3.62–16.74) had a significant association with poststroke infection and hence are independent predictors (*p* < 0.05; Table 2). Conversely, stroke type, sex, co‐morbid conditions and the use of immunosuppressive medications, beta‐blockers, prestroke antibiotics and NGT were not independently associated with infection risk (Table 2).

**Table 2:** Multivariable logistic regression model of risk factors associated with infection after acute stroke

	OR (95% CI)	*p‐*Value
Stroke type	1.17 (0.50–2.73)	0.72
Age	1.04 (1.01–1.07)	0.01*
Sex (Female)	0.84 (0.43–1.64)	0.62
NIHSS	1.05 (1.01–1.08)	0.02*
Co‐morbidities	1.13 (0.58–2.23)	0.72
Use of beta‐blockers	1.48 (0.77–2.82)	0.24
Use of immune‐suppressants	1.59 (0.54–5.16)	0.37
Use of antibiotics prestroke	1.70 (0.38–7.65)	0.11
Use of NGT	2.18 (0.84–5.64)	0.11
Use of IDC	7.79 (3.62–16.74)	<0.001*

Abbreviations: CI, confidence interval; IDC, indwelling urethral catheter; NGT, nasogastric tube; NHISS, National Institutes of Health Stroke Scale; OR, odds ratio.

* *p* < 0.05 values indicate statistical significance.

### 2.2 Advanced age exacerbates lung infection in poststroke mice

In this study, we chose to use 12‐ to 15‐month‐old mice for modelling advanced age and denoted this group as “older.” Significant mortality rate (44%) after stroke was recently reported with the use of animals >18 months old (Ritzel et al., 2018), as such we selected 12‐ to 15‐month‐old mice to represent our “older” group in order to avoid studying survival bias.

**Figure 1:**
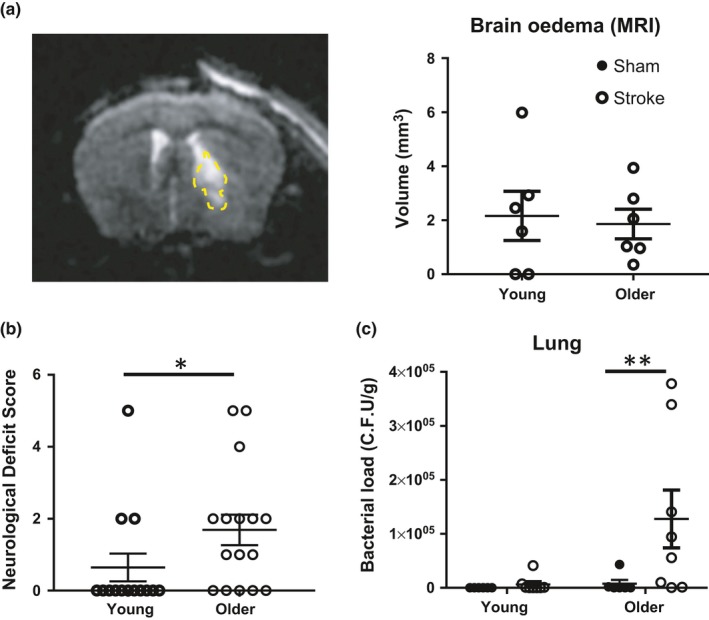
Advanced age exacerbates neurological impairment and lung infection poststroke. The following assessments were performed 24 hr after mid‐cerebral artery occlusion induction on young (7–10 weeks) and older (12–15 months) mice: (a) preclinical magnetic resonance imaging (MRI) to assess volume of brain oedema (*n* = 6/group). Oedema region indicated within yellow outline; (b) neurological assessment (*n* = 14–16/group); (c) bacteriological analysis of lung homogenates to assess poststroke lung infection (*n* = 6–8/group). Data represent the mean ± *SEM*. Significance was determined by Mann–Whitney *U* test, and a *p*‐value ≤0.05 was considered statistically significant: *p ≤ 0.05, **p ≤ 0.01

To assess the effect of advanced age on injury lesion development after the mild model of stroke, we performed preclinical magnetic resonance imaging (MRI) on our young and older cohort of animals at 24 hr following stroke onset. We observed similar oedema volumes after stroke between the two groups of mice (Figure 1a), but mice in the older group showed enhanced neurological impairment compared with younger counterparts (Figure 1b). This is consistent with the well‐reported clinical observations that elderly patients often present with greater neurological impairment and more debilitating outcomes after stroke. To understand how advanced age contributes to the development of poststroke infection, we quantified the amount of culturable bacteria from lung homogenates 24 hr after stroke. Older mice demonstrated a 100‐fold increase in culturable bacteria after stroke compared with sham‐operated controls and young counterparts, suggesting an impairment of antimicrobial defence in advanced age after stroke (Figure 1c). Levels of culturable bacteria in the blood, liver, mesenteric lymph node (MLN) and spleen remained similar and largely undetectable for both older and young mice after stroke when compared to their respective sham‐operated animals (Figure S1).

### 2.3 Older mice exhibit greater colonic permeability poststroke

**Figure 2:**
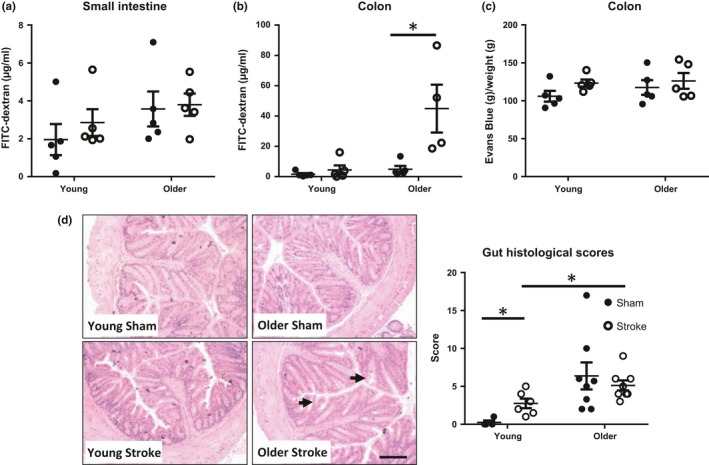
Colonic permeability increases with age after stroke. Serum levels of orally gavaged fluorescein‐isothiocyanate‐labelled (FITC)‐dextran were quantified from mid‐cerebral artery occlusion (MCAO) or sham mice to examine permeability of the (a) small intestine and (b) colon. (c) Extravasation of Evans blue dye from the colon was quantified 24 hr after MCAO or sham surgery as an indicator of vascular permeability. (d) Representative H&E images of colonic crypts at 100× magnification (scale bar = 2 mm). Histological scores for various parameters were totalled to indicate the degree of colonic pathology. A higher score indicates more visible clinical signs of colonic damage (*n* = 4–8/group). Black arrows denote examples of crypt damage and loss of architecture. Data represent the mean ± *SEM*. Significance determined by Mann–Whitney *U* test. A *p*‐value ≤0.05 was considered statistically significant: *p ≤ 0.05

Our previous study using a more severe model of transient stroke in young mice (7–10 weeks old) showed that poststroke infection is attributable to intestinal dysfunction and the subsequent systemic dissemination of host intestinal bacteria (Stanley et al., 2016). However, we are yet to understand whether age contributes to this phenomenon. To examine the effect of advanced age on gut permeability, we assessed the translocation of orally gavaged fluorescein‐isothiocyanate‐labelled dextran (FITC‐dextran) into the bloodstream. While no difference in small intestine permeability was observed in this mild model of stroke (Figure 2a), colonic permeability was significantly increased exclusively in older mice after stroke compared with their sham‐operated controls (Figure 2b). This rise in colonic permeability was not accompanied by changes in vascular permeability as determined by the detection of Evans blue dye in intestinal tissue (Figure 2c). Furthermore, while older poststroke mice did not demonstrate visible clinical signs of colonic damage (i.e., stool inconsistency or faecal occult blood) compared with sham‐controls, older animals clearly displayed worse colonic inflammation and injury microscopically than their young counterparts as assessed by histology in a blinded manner (Figure 2d).

### 2.4 Older mice show evidence of colonic barrier breakdown poststroke

**Figure 3:**
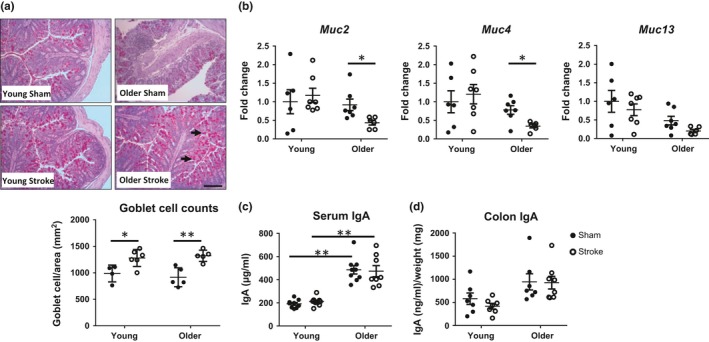
Stroke induces robust mucosal changes in older animals. (a) Representative images of colonic goblet cells at 100× magnification (scale bar = 2 mm; black arrow), stained using PAS‐Alcian blue at 24 hr postsurgery (*n* = 4–5/group). (b) Gene expression of mucin 2 (*Muc2*), *Muc4* and *Muc13* from the colon of young and older mice was analysed by qPCR 24 hr after mid‐cerebral artery occlusion (MCAO) and expressed as a fold change relative to that of young sham‐operated controls (*n* = 7/group). (c) Protein levels of secretory IgA in the serum and (d) colon 24 hr after MCAO or sham surgery (*n* = 7–8/group). Data represent the mean ± *SEM*. Significance was determined by one‐way ANOVA with post hoc comparison and Holm–Sidak multiple testing correction. A *p*‐value ≤0.05 was considered statistically significant: *p ≤ 0.05, **p ≤ 0.01

Integrity of the colonic barrier encompasses many structural and physiochemical aspects that collaborate to regulate intestinal permeability and prevent bacterial translocation (Chelakkot, Ghim, & Ryu, 2018). The intestinal mucosal layer consists of mucin produced by goblet cells, together with secretory IgA, which form the crucial first line of defence. At 24 hr after stroke, there was a significant increase in goblet cell numbers in the colon of both young and older mice compared with their respective sham‐operated controls (Figure 3a). Interestingly, gene expression of key components that form the mucus layer, mucin 2 (*Muc2*), mucin 4 (Muc4) and mucin 13 (*Muc13*) was decreased only in the older mice, but not in young mice, after stroke (Figure 3b). This reduction in mucin expression suggests a potential age‐dependent breakdown of the mucus barrier following stroke onset. Conversely, the levels of secretory IgA in the serum and colon remained unaltered after stroke (Figure 3c,d). However, it was noted that serum IgA expression was elevated in older animals independent of stroke when compared to younger counterparts (Figure 3c).

### 2.5 Reduced tight junction protein expression in older mice after stroke

**Figure 4:**
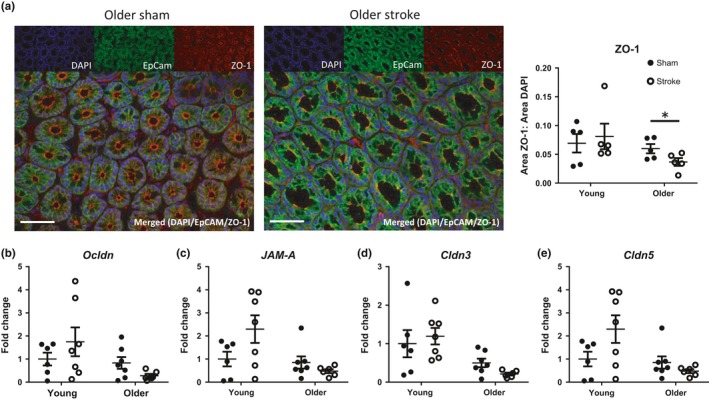
Age‐dependent disruption of colonic tight junctions after stroke. (a) Representative immunofluorescence images of colonic cross sections at magnification of 400× (scale bar = 50 µm), stained for nuclei (DAPI; blue), EpCAM (Alexa Fluor 488; green) and ZO‐1 (Alexa Fluor 568; red). The area of ZO‐1 staining relative to DAPI staining was quantified 24 hr after mid‐cerebral artery occlusion (MCAO) or sham surgery (*n* = 5/group). The gene expression of (b) occludin (*Ocldn*), (c) junctional adhesion molecule‐A (JAM‐A), (d) claudin 3 (*Cldn3*) and (e) claudin 5 (*Cldn5*) from the colon of young and older mice was analysed by qPCR 24 hr after MCAO and expressed as a fold change relative to that of young sham‐operated controls (*n* = 7/group). Data represent the mean ± *SEM*. Significance was determined by unpaired *t* test. A *p*‐value ≤0.05 was considered statistically significant: *p ≤ 0.05

Beyond the first line of defence, the colonic barrier is strengthened further by important multi‐complex tight junctions that ensure strong scaffolding of intestinal epithelial and endothelial cells (Chelakkot et al., 2018). Alterations in the protein expression of tight junctions can lead to intestinal structure disruption and changes to paracellular and intercellular barrier permeability. Using immunofluorescence staining, we observed that the expression of key proteins of the tight junction complex, zonula occludens‐1 (ZO‐1), was reduced exclusively in the colon of older mice after stroke, and not their young counterparts (Figure 4a). This pattern of reduced expression appeared specific to ZO‐1 as it was not statistically different across gene expressions of other prominent tight junctions in the colon or small intestine of older poststroke mice: occluden (*Ocldn*), junctional adhesion molecule (*JAM‐A*), claudin 3 (*Cldn3*) and claudin 5 (*Cldn5*) (Figure 4b–e and Figure S2).

### 2.6 Tumour necrosis factor‐α facilitates the breakdown of colonic barriers poststroke

**Figure 5:**
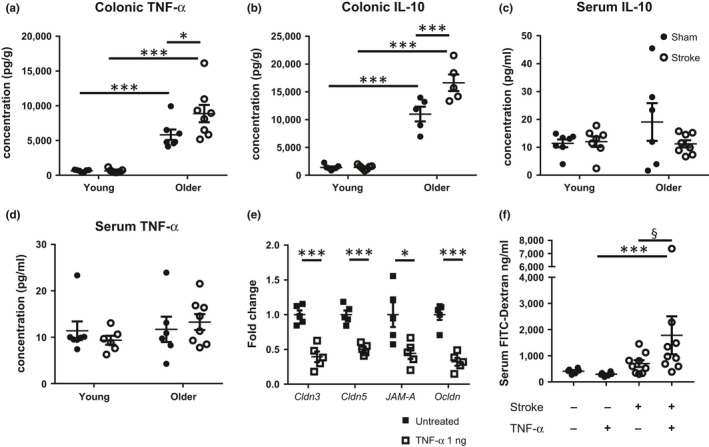
Tumour necrosis factor (TNF)‐α may facilitate the breakdown of colonic barriers poststroke. Protein expression of TNF‐α and IL‐10 in the (a–b) colon and (c–d) serum from young and older mice was quantified 5 hr after mid‐cerebral artery occlusion (MCAO) or sham surgery (*n* = 5–8/group). (e) Colon cross sections from naïve older mice were treated with recombinant TNF‐α for 1 hr in an ex‐vivo setting, while untreated tissues acted as controls. Gene expression of claudin 3 (*Cldn3*), claudin 5 (*Cldn5*), junctional adhesion molecule‐A (*JAM‐A*) and occludin (*Ocldn*) was assessed by qPCR and expressed as a fold change relative to untreated older colons (*n* = 5/group). (f) To examine whether TNF‐α can alter intestinal permeability in vivo, recombinant TNF‐α (20 µg/kg) or saline as control was administered (i.p.) immediately following blood reperfusion to young MCAO and sham‐operated mice. Serum levels of orally gavaged FITC‐dextran were quantified to examine colon permeability. Data represent the mean ± *SEM*. Significance was determined by one‐way ANOVA with post hoc comparison and Holm–Sidak multiple testing correction. A *p*‐value ≤ 0.05 was considered statistically significant: *p ≤ 0.05, ***p ≤ 0.001 and ^§^ *p* ≤ 0.1 considered a significant trend

Given that older mice showed greater histological evidence of intestinal inflammation than their younger counterparts (Figure 2d), we hypothesized that the stroke‐induced pro‐inflammatory microenvironment promotes barrier breakdown in the colon of older mice. Indeed, previous studies have reported that pro‐inflammatory cytokines, specifically tumour necrosis factor (TNF‐α), play a critical role in regulating tight junction proteins in a myosin light chain kinase (MLCK)‐dependent manner, inducing intestinal permeability, pathology and inflammaging (Shen, 2012; Yu et al., 2010). In this study, expression of TNF‐α and IL‐10 was elevated exclusively in the colonic tissue of older, and not young mice 5 hr poststroke compared with sham‐operated cohorts (Figure 5a,b): a time point that preceded any changes in tight junction expression of *Cldn3*, *Cldn5*, *Ocldn* and ZO‐1 (Figure S3). Additionally, we found no difference in the total number of CD45^+^ leucocytes, CD3^+^ T cells, CD11b^+^ myeloid cells, Ly6G^+^/Ly6C^−^ neutrophils or Ly6G^−^/Ly6C^+^ monocytes in the colon, suggesting this early pro‐inflammatory cytokine environment was not be driven by cellular immune changes (Figure S4). By 24 hr poststroke, elevated TNF‐α and IL‐10 expression in the colon of older poststroke mice returned back to baseline sham levels (Figure S5A,B). The earlier acute pro‐inflammatory response appears specific to the colon as circulating serum levels of TNF‐α and IL‐10 remain unchanged at both the 5‐ and 24‐hr time point following stroke (Figure 5c,d and Figure S5C,D).

Tumour necrosis factor‐α signalling has been shown to participate in cerebral neuroinflammation after stroke (Hallenbeck, 2002); thus, the use of anti‐TNF‐α or recombinant TNF‐α to examine its role in stroke‐induced gut dysfunction in vivo may also impact brain infarct development. Therefore, to firstly confirm the critical role of TNF‐α with advancing age on the regulation of tight junction complexes independent of brain ischaemic alterations as a confounding factor, we adopted an ex‐vivo strategy. Cross sections of colon from naïve older mice were treated with recombinant TNF‐α for 1 hr in an ex‐vivo setting to mimic the induction of stroke‐induced pro‐inflammatory responses, and changes in gene expression of various tight junctions assessed. We found that TNF‐α treatment significantly reduced colonic gene expression of *Cldn3*, *Cldn5*, *JAM‐A* and *Ocldn* compared to untreated colons (Figure 5e), suggesting disruption of intestinal barriers. In separate experiments, to examine the contribution of TNF‐α in facilitating gastrointestinal permeability in an in vivo stroke setting, we administered recombinant TNF‐α (or saline i.p. as control) to young sham‐operated and poststroke mice, and assessed FITC‐dextran translocation into the bloodstream. It is important to note that both of these groups with young mice did not show evidence of intestinal dysfunction without the treatment of TNF‐α (Figure 2b). In the setting of stroke, administration of TNF‐α induced greater gastrointestinal permeability as indicated by a trend towards increased serum levels of orally gavaged FITC‐dextran compared to their corresponding saline‐treated mice (Figure 5f). Importantly, the ability of TNF‐α to regulate intestinal permeability is dependent on the induction of stroke as sham‐operated young mice with TNF‐α treatment were unaffected (Figure 5f). Taken together, our data suggest that early production of TNF‐α localized in the colon in older poststroke mice can robustly down regulate the expression of a number of tight junction proteins and contribute to the breakdown of colonic barriers.

### 2.7 Enhanced bacterial translocation in older mice after stroke

**Figure 6:**
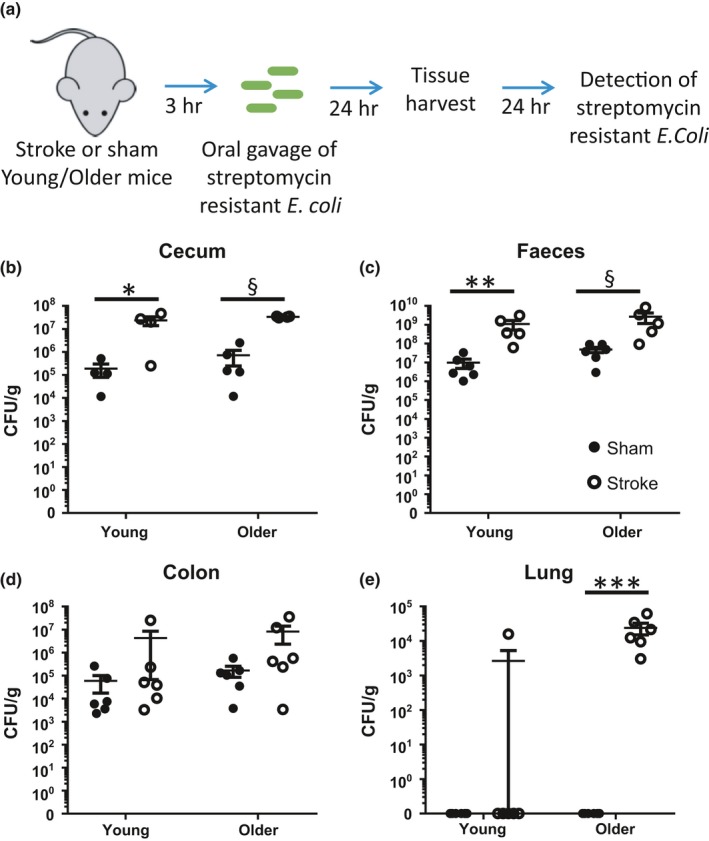
Stroke results in the translocation of intestinal bacteria for systemic dissemination in older animals. (a) Three hours after mid‐cerebral artery occlusion (MCAO) or sham surgery, young and older mice were orally inoculated with streptomycin‐resistant derivative of the *Escherichia coli* strain DLL206, at a time point when gut permeability was evident. The (b) caecum, (c) faeces, (d) colon and (e) lung were assessed 24 hr later for the presence and load of streptomycin‐resistant *E. coli*. *n* = 5–6/group. Data represent the mean ± *SEM* (log‐scale). Significance was determined by one‐way ANOVA with post hoc comparison and Holm–Sidak multiple testing correction. A *p*‐value ≤0.05 was considered statistically significant: *p ≤ 0.05, **p ≤ 0.01, ***p ≤ 0.001 and ^§^ *p* ≤ 0.1 considered a significant trend

With various components of the intestinal barrier showing dysfunction and enhanced gut inflammation after stroke in older mice, we next assessed if these components in combination can promote the translocation and systemic dissemination of colonic bacteria. We orally gavaged a streptomycin‐resistant derivative of an *E. coli* strain to young and older mice 3 hr after stroke or sham surgery, a time point when gut permeability was evident. The lungs, faeces, caecum, colon and various sections of the small intestine (duodenum, jejunum, ileum) were assessed for the presence of streptomycin‐resistant *E. coli*, 24 hr postinoculation (Figure 6a). Both young and older mice had significantly higher levels of streptomycin‐resistant *E. coli* in the caecum and faeces after stroke onset (Figure 6b,c). Conversely, detectable levels of streptomycin‐resistant *E. coli* remained unchanged in the colon, duodenum, jejunum and ileum after stroke (Figure 6d and Figure S6). Importantly, we noted that while all older mice after stroke had detectable levels of streptomycin‐resistant *E. coli* in their lungs, these bacteria remained almost undetectable in sham‐operated controls and young counterparts (Figure 6e). Therefore, the results of this inoculation experiment strongly suggest the stroke‐induced colonic inflammation and permeability observed in the older mice may contribute to the translocation of commensal bacteria for systemic dissemination.

## 3 DISCUSSION

Aside from neurological damage, bacterial pneumonia is the most frequent severe complication, and the most common cause of death, in patients with stroke (Meisel et al., 2005; Shim & Wong, 2018). Confirming previous clinical studies, we showed using a retrospective patient cohort that increasing age is an independent predictor for developing infection after stroke. Further to this, we utilized an experimental model of mild transient ischaemic stroke to examine how advance age may render the host more susceptible to poststroke infection. The present study demonstrated that the frequency and severity of poststroke infections are greater in older animals compared with their younger counterparts. This age‐dependent onset of poststroke pneumonia is attributed to increased colonic inflammation, permeability, structural breakdown and bacterial translocation to peripheral tissues such as the lung, where they may become pathogenic to worsen overall disease outcomes.

A growing number of experimental and clinical studies suggest that altered signalling between the brain, gut and microbiota is implicated in several neurological disorders, including autism, Parkinson's disease, Alzheimer's and stroke (Sharon, Sampson, Geschwind, & Mazmanian, 2016; Stanley, Moore, & Wong, 2018). In fact, gastrointestinal dysfunction is a common complication in patients with head injuries (Pilitsis & Rengachary, 2001). This is unsurprising since the central nervous system (CNS) modulates the highly innervated gastrointestinal tract by branches of the autonomic nervous system and the hypothalamic/pituitary/adrenal (HPA) axis (Rao & Gershon, 2016). In the setting of stroke, up to 50% of patients experience pathophysiological changes in the gastrointestinal tract, including dysphagia, gastrointestinal bleeding and constipation (Camara‐Lemarroy, Ibarra‐Yruegas, & Gongora‐Rivera, 2014). Gastrointestinal complications after stroke are associated with overall poor patient outcomes, including delayed patient recovery times, increased mortality rates and deteriorating neurologic function (Camara‐Lemarroy et al., 2014; Paciaroni et al., 2004). Indeed, the findings from our current study indicate that advanced age exacerbates the risk of gastrointestinal dysfunction after mild brain injury. Despite similar brain oedema volumes between young and older mice after stroke, only older animals demonstrated rapid colonic alterations within 24 hr after stroke. The colon of older stroke mice showed significant breakdown of barriers that help maintain gut integrity, including reduced expression of mucin and tight junction proteins. This breakdown was sufficient to allow the translocation of orally inoculated bacteria (streptomycin‐resistant *E. coli*) from the gut to peripheral tissues. While we cannot confirm from our clinical and experimental studies that stroke‐induced spontaneous lung infection results directly from translocated host gut microbiota, one may speculate that it is a possible cause. Supporting this idea, we have previously shown that the majority (>70%) of bacteria detected in patients with stroke at a median age of 76.5 who developed infections were common commensal bacteria that normally reside in the intestinal tract (*Enterococcus* spp.,* E. coli* and *M. morganii*; Stanley et al., 2016). Further studies to examine whether only select bacterial types from the colon are capable of translocation, survival and expansion in peripheral tissues, such as the lung, poststroke are required. Additionally, examining how this selectivity changes with increasing age would be important in the quest for novel therapies independent of antibiotics.

Previous work has shown that significant adverse effects of normal aging, such as chronic inflammation and decline in innate immune function, can impend on events critical to recovery after stroke and contribute to poor prognosis in the elderly (Crapser et al., 2016; Ritzel et al., 2018). We additionally show in the present study that excessive induction of pro‐inflammatory responses localized in the gut can disrupt intestinal function and may contribute to worse stroke outcomes for older individuals. Indeed, we showed that the compromised intestinal barriers following stroke in older animals were associated with the early and increased production of pro‐inflammatory TNF‐α. This is consistent with previous studies showing that anti‐TNF therapy reverses the negative effects of age‐associated inflammation and microbial dysbiosis on intestinal permeability and bacterial translocation (Thevaranjan et al., 2017). The mechanistic regulation of TNF‐α on tight junction proteins such as ZO‐1 is highly complex, but morphological studies using cell lines and in vivo imaging techniques have shown its interaction and resulting response can happen quickly. It is believed TNF‐α interacts with ZO‐1 to induce cytoskeletal reorganization via modulation of myosin light chain kinase (MLCK) activity, resulting in changes to the perijunctional actomyosin ring structure critical for cell anchorage (Yu et al., 2010). TNF‐α can additionally induce occludin removal through caveolar endocytosis to affect structural and functional tight junction regulation (Marchiando et al., 2010). The likely source of elevated TNF‐α in the colon of older poststroke mice is the damaged intestinal epithelium, as the immune composition of the gut and serum concentrations of this pro‐inflammatory cytokine remained unchanged after stroke. In fact, TNF‐α production by intestinal epithelial cells has been shown to be key in initiating pathology in Crohn's disease (Roulis, Armaka, Manoloukos, Apostolaki, & Kollias, 2011). Therefore, specific inhibition of TNF‐α production by the intestinal epithelium may present a new strategy to reduce gut permeability and subsequently gut‐derived bacterial lung infection after stroke.

We have previously shown that young mice subjected to a more severe model of transient stroke resulted in dysfunction of the small intestine but not the colon (Stanley et al., 2016). The subsequent systemic dissemination of gut‐derived bacteria from the small intestine was driven by sympathetic neural signalling as pharmacological administration of β‐adrenergic receptor inhibitors (propranolol or metoprolol) restored stroke‐induced ileum dysfunction and reduced poststroke infection. Another study using multiple experimental stroke models also showed that only large stroke lesions cause barrier dysfunction, reduced motility and microbiota dysbiosis of the small intestine in younger mice (Singh et al., 2016). It is feasible that the degree of intestinal dysfunction and location resulting from stroke is dependent on a variety of factors highly influenced by the process of aging: intestinal regeneration and repair, sympathetic drive, gut inflammatory state, immune composition and function, and the existing host microbiota. Indeed, our study showed that older animals displayed worse colonic inflammation and injury microscopically prior to stroke induction when compared to younger counterparts, indicating innate biological differences with age. Consistent with these findings, a recent study demonstrated that older mice showed increased apoptotic cells in intestinal crypts and villi, with functional impairment of isolated intestinal epithelial stem cells, suggesting a reduced capacity for tissue repair following injury (Moorefield et al., 2017).

Given that the gut barrier integrity and intestinal inflammatory balance are closely linked with gut microbiome, it is possible that age‐dependent alterations in the microbiota further influence stroke outcomes. It is known that older individuals have a very different gut microbiota profile compared to healthy adults. Generally, the gut microbiome of elderly individuals is characterized by reduced bacterial diversity with a shift towards lower levels of beneficial populations such as bifidobacteria, and this is associated with increased frailty (Jackson et al., 2016; Nagpal et al., 2018). The mechanism of microbiota change with aging is not well understood, but it is thought that factors associated with cell senescence, immune changes, co‐morbidities, intestinal physiology and lifestyle play a role. Several recent studies using high‐throughput gene sequencing tools demonstrated that stroke induces robust changes to the intestinal mucosal microbiota, with an overall reduction in species diversity (Singh et al., 2016; Stanley et al., 2018). Furthermore, it is important to remember that brain‐gut communication is bidirectional, with emerging evidence showing that disruption of microbial‐host symbiosis prior to stroke also leads to altered infarct severity after cerebral ischaemia (Benakis et al., 2016).

A better understanding of age‐associated alterations in mucosal microbiota composition, and their reciprocal impact on the host biological pathways, will assist in designing novel and targeted therapeutic approaches to reduce neurological damage and infectious complications after stroke.

## 4 EXPERIMENTAL PROCEDURES

### 4.1 Patient sampling, data evaluation and analysis

A retrospective cohort design was used, where all information was obtained from scanned medical and electronic records available at the time of data collection. All patients primarily admitted for an acute stroke to Monash Medical Centre between 16 January 2015 and 10 February 2016 were enrolled and the data present here is a subset of a recent published clinical study (Phan et al., 2019). Multivariable logistic regression was used to determine the association between stroke and the presence of infection after adjusting for confounding factors. The patient retrospective study was approved by Monash Health Human Research Ethics Committee. See Data S1 for details on patient sampling, data evaluation and analysis.

### 4.2 Mice

Young (7–10 weeks) and older (12–15 months) male C57BL/6J mice were obtained and housed by the Monash Animal Research Platform (MARP) under specific‐pathogen‐free (SPF) conditions. All naïve young mice were housed in groups of no more than five animals in each cage after weaning, while older mice were individually housed. Water and food pellets (Irradiated Rat and Mouse, Specialty Feeds, Australia) were provided ad libitum, and cages were changed weekly. All animal procedures were conducted in accordance with Australian National Health and Medical Research regulations on the use and care of experimental animals, and approved by the Monash University Animal Ethics Committee (MMCB/2016/10).

### 4.3 Mouse focal cerebral ischaemia model

The mid‐cerebral artery occlusion (MCAO) model was performed as previously described (Nicholls, Wen, Hall, Hickey, & Wong, 2018; Stanley et al., 2016, 2018). Young and older animals were given 20 min of MCAO followed by reperfusion to model a mild form of ischaemic stroke, resulting in <10% mortality in both groups to avoid survival bias in our study. Sham‐operated animals underwent anaesthetic, neck incision and artery isolation only. All animals were individually housed after MCAO or sham surgery. See Data S1 for surgical details.

To examine whether TNF‐α can alter intestinal permeability in vivo, recombinant TNF‐α (20 µg/kg) or saline as control was administered (i.p.) immediately following blood reperfusion to young stroke and sham‐operated mice.

### 4.4 Magnetic resonance imaging

Oedema volumes were measured using MRI 24 hr after MCAO in young and older mice. Measurements of oedema exclude the volume of the ventricle at the dorsal section of the infarct brain hemisphere. See Data S1 for MRI scanning procedure and settings.

### 4.5 Neurological assessment

At 24 hr after MCAO, neurological assessment was performed on young and older mice using an established six‐point scoring system (Kim et al., 2014). See Data S1 for scoring parameters.

### 4.6 Gut permeability assay

Mice were orally gavaged with 500 mg/kg of 4.4‐kDa fluorescein‐isothiocyanate‐labelled dextran (FITC–dextran; Sigma) at 2 hr after MCAO. Animals were anesthetized with isoflurane at 1 hr (small intestine permeability) or 4 hr (colon permeability) after gavage, and a cardiac puncture performed to collect serum. Serum concentrations of FITC–dextran were determined relative to a standard curve (top standard at 1,250 µg/ml) at an excitation of 485 nm.

### 4.7 Vascular permeability assay

At 24 hr after MCAO, animals were given an intravenous injection of 4 ml/kg 2% Evans blue (Sigma) in saline. Mice were then transcardially perfused with saline at 4 hr after Evans blue injection. The whole colon was homogenized in 3 ml of *N*,*N*‐dimethylformamide (Sigma) and further incubated in *N*,*N*‐dimethylformamide overnight at 55°C. Supernatant containing Evans blue was collected by centrifugation at 500 *g* for 10 min, and concentrations of Evans blue determined relative to a standard curve (top standard at 1,000 µg/ml) at an excitation of 620 nm.

### 4.8 Colon histological scoring

At 24 hr after MCAO, a section of the distal colon was collected for haematoxylin and eosin (H&E) staining. For each sample, two representative images of colonic crypts at 100× magnification were captured using the Leica DM LB widefield microscope and MC120 HD camera (Leica) for histological scoring in a blinded manner. Parameters for histology scoring were previously established (Shen et al., 2018) and detailed in Data S1. Total overall score indicates the degree of colonic pathology after MCAO or sham operation.

### 4.9 Periodic acid‐Schiff staining

At 24 hr after MCAO, a section of the distal colon was collected for Periodic acid‐Schiff reagent (PAS) staining according to standard protocols. For each sample, two representative images of colonic crypts at 100× magnification were captured using the Leica DM LB widefield microscope and MC120 HD camera (Lecia). The number of goblet cells per colonic crypt was quantified and presented as number of cells per mm^2^.

### 4.10 RNA isolation and RT–qPCR

qRT–PCR was performed with standard protocols (Nicholls et al., 2018) using the Power SYBR Green PCR Master Mix (Applied Biosystems), targeting expression of *18S*, occludin, claudin 3, claudin 5, junctional adhesion molecule‐A, mucin 2, mucin 4 and mucin 13. Primer sequences are detailed in Table S3. Data were normalized to housekeeping gene *18S* and analysed using the 2^(−ΔΔC^ *^t^* ^)^ method. Gene expression was expressed as fold change relative to colon tissue from sham‐operated animals.

### 4.11 Immunofluorescence staining

OCT‐embedded colon sections were stained for the expression of ZO‐1 (1:100; ThermoFisher 61‐7300), CD32 (EpCAM, 1:500; ThermoFisher 14‐5791‐81) and DAPI (Sigma 10236276001). See Data S1 for detailed staining protocol. ZO‐1 expression was quantified using representative images taken on the Nikon C1 Invert confocal microscope (Nikon) at a magnification of 400×. Images were captured using a pattern which ensured unbiased selection and no overlap. Approximately 6–8 images were captured for each colon section for analysis and averaged. imagej (NIH) was used for image processing to quantify the area of ZO‐1 respective to area of DAPI staining.

### 4.12 Inoculation of bacteria

To investigate the potential translocation of bacteria after stroke, 10^10^ colony‐forming units (c.f.u.) of the streptomycin‐resistant *E. coli* strain DLL206 was inoculated into mice via oral gavage 3 hr after sham or MCAO surgery. The detection of streptomycin‐resistant *E. coli* from various tissues (lung, duodenum, jejunum, ileum, caecum and faecal) was performed 22 hr later by plating tissue homogenates on 2YT agar plates containing 100 μg/ml streptomycin.

### 4.13 Bacteriological analysis

At the time of cull, mice were washed with 70% ethanol under sterile conditions. Lungs, duodenum, jejunum, ileum, caecum and/or faecal pellet were collected and homogenized in sterile PBS. For determination of colony‐forming units (c.f.u.), tissue homogenate was serially diluted in PBS and plated onto brain–heart infusion (BHI) agar plates supplemented with 5% sheep blood (for determination of bacterial load in poststroke mice) or 2YT agar plates containing streptomycin (for *E. coli* inoculation experiments), incubated at 37°C for 18 hr and counted to quantify the number of streptomycin‐resistant *E. coli* colonies.

### 4.14 Enzyme‐linked immunosorbent assay

Protein concentrations of IgA, tumour necrosis factor (TNF)‐α and interleukin (IL)‐10 in colonic tissue were measured using the Bethyl Laboratories IgA enzyme‐linked immunosorbent assay (ELISA) Kit (Bethyl Laboratories), Mouse TNF ELISA Set II (BD Biosciences; Cat.558534) and Mouse IL‐10 ELISA Set (BD Biosciences; Cat.555252) respectively, according to the manufacturer's instructions.

### 4.15 Cytometric bead array

Serum cytokine levels of TNF‐α, IL‐10 and IL‐6 were measured using the BD Cytometric bead array (CBA) Mouse Th1/Th2/Th17 Cytokine Kit (BD Biosciences) according to the manufacturer's instructions. The LSR‐Fortessa (BD Biosciences) and FlowJo (Tree Star) were used for acquisition/analysis.

### 4.16 Flow cytometry of colon leucocytes

Single‐cell suspensions for flow cytometry were prepared as previously described (Shen et al., 2018) and detailed in Data S1. Leucocytes were stained with CD45‐PE (30‐F11; eBioscience), CD11b‐FITC (M1/70; BioLegend), CD3‐APC (145‐2C11; BD Biosciences), Ly6C‐APC Cy7 (AL‐21; BD Biosciences) and Ly6G‐BV510 (1A8; BioLegend), together with Fc receptor blocking using anti‐CD16/32 before resuspension in FACS buffer containing 2% FBS and viability dye (7‐aminoactinomycin D; 7‐AAD). The LSR‐Fortessa (BD Biosciences) and FlowJo (Tree Star) were used for acquisition/analysis.

### 4.17 Ex‐vivo assessment of TNF‐α on colonic tight junction complexes

Colons of naïve older (12–15 months) male C57BL/6J mice were carefully excised, dissected into 1 cm cross sections, faecal matter gently removed and placed in carbonated Krebs buffer supplemented with 2 g/L d‐glucose. Carbonated Krebs buffer is considered the most biorelevant buffer system for the simulation of intestinal conditions (Fadda, Merchant, Arafat, & Basit, 2009). Colon cross sections were submerged into 1 ml of Krebs buffer (1 ml) and treated for a total of 1 hr with either 0 or 1 ng of recombinant mouse TNF‐α (BioLegend). During treatment, the Krebs buffer and colon sections were constantly carbonated at the right pH to ensure tissue viability. Following treatment, tissues were processed for qRT–PCR to examine the role of TNF‐α on tight junction complex expression.

### 4.18 Statistics

Quantitative data for experimental mouse studies are presented as mean ± standard error of the mean (*SEM*). Statistical analyses were conducted using graphpad prism Software. Data sets were tested for normality using the Shapiro–Wilk normality test. Nonparametric data were analysed using the Mann–Whitney *U* test. Comparisons of multiple parametric data sets were analysed using one‐way analysis of variance (ANOVA) with post hoc comparison with Holm–Sidak multiple testing correction. Single comparisons of parametric data sets were analysed using the Student *t* test. Adjusted *p*‐value ≤0.05 was considered statistically significant.

## CONFLICT OF INTEREST

None declared.

## AUTHORS CONTRIBUTION

S.W.W. and C.H.Y.W conceived and designed the experiments, and contributed to manuscript writing. S.W.W. carried out all animal experiments and analysed all experimental results with assistance from: R.S. (MCAO surgery), B.J.W (ELISA and RNA extraction), Y.N.S. (*E. coli* inoculation experiments), K.P.K (gut histology scoring), A.J.N. (RNA extraction), S.S (flow cytometry), T.S. (MRI), M.de V. (MRI), D.L. (*E. coli* inoculation experiments) and C.H.Y.W (MCAO surgery). L.H., V.K.S., H.M. and T.G.P. carried out the patient study and data analysis. All authors carried out manuscript revision.
