# Supplementary material for: Advanced age promotes colonic dysfunction and gut‐derived lung infection after stroke
Source: Aging Cell. 2019 Jun 14;18(5):e12980. doi: 10.1111/acel.12980 (PMC6718525; doi:10.1111/acel.12980)
Supplement: Supplementary file 1 [file ACEL-18-e12980-s001.docx]

**Advanced age promotes colonic dysfunction and gut-derived lung infection after stroke**

Shu Wen Wen^1^, Raymond Shim^1^, Luke Ho^1,2^, Brooke J. Wanrooy^1^, Yogitha N. Srikhanta^3^, Kathryn Prame Kumar^1^, Alyce J. Nicholls^1^, SJ Shen^1^, Tara Sepehrizadeh^4^, Michael de Veer^4^, Velandai K. Srikanth^2^, Henry Ma^5^, Thanh G. Phan^5^, Dena Lyras^3^ and Connie H. Y. Wong^1 *^

^1^ Centre for Inflammatory Diseases, Department of Medicine, School of Clinical Sciences, Monash University, Clayton, Victoria 3168, Australia.

^2^ Department of Medicine (Academic Unit), Peninsula Clinical School, Central Clinical School, Monash University, Frankston, Victoria, Australia.

^3^Monash Biomedicine Discovery institute, Department of Microbiology, Monash University, Clayton, Victoria 3800, Australia.

^4^Monash Biomedical Imaging, Monash University, Clayton, Victoria 3800, Australia.

^5^Stroke and Ageing Research Group, Department of Medicine, School of Clinical Sciences, Monash Medical Centre, Monash University, Clayton, Victoria, Australia.

^*^Address for correspondence: Connie H. Y. Wong, PhD., Centre for Inflammatory Diseases, Department of Medicine, School of Clinical Sciences at Monash Health, Monash University, Clayton, VIC 3168 Australia; Email: [connie.wong@monash.edu](mailto:connie.wong@monash.edu)

**Supplemental Information Data S1**

**Supplemental Experimental Procedures**

*Patient sampling*

A retrospective cohort design was used, where all information was obtained from scanned medical and electronic records available at the time of data collection. All patients primarily admitted for an acute stroke to Monash Medical Centre between 16^th^ January 2015 and 10^th^ February 2016 were enrolled. The patient retrospective study was approved by Monash Health Human Research Ethics Committee. A waiver of individual consent was granted given that the study was retrospective in nature and there was no intervention component to this study.

Patients with transient ischemic attacks and those who developed a stroke as a complication of hospitalisation and thus had a separate reason for admission, were excluded. Included patients were followed-up for development of infections until the day of discharge from the acute ward or till death, depending on which came earlier. If death occurred in the sub-acute setting (rehabilitation unit) within the same admission, it was also recorded. All data extraction was performed by a single person (L.H.). The severity of stroke was determined in most cases using the National Institute of Health Stroke Scale (NIHSS) within 24 hrs of admission. If that was not documented, a retrospective NIHSS was obtained on the documented signs and symptoms on admission. Computed tomography (CT) brain scans at stroke onset were used to differentiate between ischemic and haemorrhagic stroke, taking care not to misclassify those with haemorrhagic transformation within an ischemic stroke. The severity of stroke was determined in most cases using the National Institute of Health Stroke Scale (NIHSS) within 24 hrs of admission. If that was not documented, a retrospective NIHSS was obtained on the documented signs and symptoms on admission.

*Patient data evaluation and analysis*

All infections that developed while patients were in the acute stroke unit were recorded. Infections were defined with reference to the Centers of Disease Control (CDC) criteria for infections, which required certain clinical, pathological, radiological and microbiological evidence as explained in **Supplementary Table 1**.

All relevant evidence relating to a potential infection was recorded, systematically reviewed by a senior physician (V.K.S.) with reference to the CDC/NHSN criteria, and classified as “Definite”, “Probable” or “Unlikely”. Definite infections were diagnosed in those with sufficient information to meet the CDC/NHSN criteria. Probable infections were diagnosed in those with insufficient information to meet the CDC/NHSN criteria, but were regarded as a likely infection by the physician. Unlikely infections were diagnosed in those with sufficient information to not meet the criteria.

Furthermore, data were collected on the use of antibiotics, and invasive procedures such as intravenous cannula (IVC), indwelling catheter (IDC), nasogastric tube (NGT), endotracheal tube (ETT). Information regarding the use of pre-admission medications that may influence immune function and infection risk including antibiotics, immunosuppressive medications and beta-blockers was obtained through pharmacy records (**Supplementary Table 2**). The presence or history of co-morbid conditions that may predispose to infection risk was also recorded.

Multivariable logistic regression was used to determine the association between stroke and the presence of infection after adjusting for confounding factors of age, sex, stroke severity, the use of medications affecting infection risk (specifically immunosuppressive including corticosteroids, immunological therapies, disease-modifying anti-rheumatic drugs, chemotherapy), the presence of any comorbid conditions affecting infection risk (such as diabetes mellitus, chronic lung disease, chronic liver disease, active malignancy), the use of indwelling urinary catheter and the presence of NGT feeding.

*Mouse focal cerebral ischemia model*

Young and older animals were given 20 min of MCAO followed by reperfusion to model a mild form of ischemic stroke, resulting in <10% mortality in both groups to avoid survival bias in our study. Briefly, mice were anesthetized by intraperitoneal (i.p.) injection with a mixture 100 mg/kg of ketamine and 10 mg/kg of xylazine, and body temperature maintained at 37 °C. An incision to the neck was made, and the common, external and internal carotid arteries dissected free. A silicon-coated monofilament of 0.21-0.25 mm was advanced from the internal carotid artery to the origin of the mid-cerebral artery (MCA) to occlude blood flow. After 20 min of occlusion, the monofilament was withdrawn to allow reperfusion. A laser Doppler perfusion monitor on the cranium of each mouse was used to verify successful occlusion of the MCA (Perimed). Following surgery, animals recovered on a 37 °C heat pad overnight. Sham-operated animals underwent anaesthetic, neck incision and artery isolation only. All animals in the study were randomly assigned to the sham-operated or MCAO-operated cohort.

*Magnetic resonance imaging*

Throughout the MRI scanning procedure, mice were anesthetized with 2% isoflurane. Mice were positioned prone, head first, in a Bruker 9.4T MRI Small Animal Scanner, with their body temperatures maintained at 37 °C. T2-weighted images were acquired using T2-turboRare sequence with the following parameters: repetition time/effective echo time, 9000/51 ms; field of view, 20 × 20 mm; image size, 128 × 128; slice thickness, 0.2 mm; echo spacing, 17 ms; average, 6; rare factor, 8. Cerebral infarct volumes were quantified using ImageJ (NIH) in a blinded manner.

*Neurological assessment*

At 24 hrs after MCAO, neurological assessment was performed on young and older mice using an established six-point scoring system (Kim *et al.* 2014): 0, normal motor function; 1, flexion of torso and contralateral forelimb when mouse is lifted by the tail; 2, circling when mouse held by the tail on a flat surface; 3, leaning to the one side at rest; 4, no spontaneous motor activity; 5, death within 24 hrs.

*Colon histological scoring*

Parameters for histology scoring: the level of tissue involvement (0: none, 1: mucosa, 2: mucosa and sub-mucosa, 3: sub-mucosa-transmural, 4: transmural), level of inflammation (0: none, 1: mild, 2: moderate, 3: severe, 4: severe with GALT involvement), involvement of crypt and epithelial (0: none, 1: surface, 2: 2/3 basal, 3: crypt and goblet cell loss, 4: crypt and goblet cell destruction with hyperplasia, surface epithelial destruction and haemorrhage), and level of lamina propria/sub-mucosa oedema (0: none, 1: mild, 2: moderate, 3: moderate-severe, 4: severe) (Shen *et al.* 2018). Scores for all parameters were totalled to achieve an overall total score that indicates the degree of colonic pathology after MCAO or sham-operation.

*Immunofluorescence staining*

At the time of cull, colon sections were butterflied open and embedded flat in OCT. OCT-embedded sections (10 µm) were air-dried, fixed in 10% neutral buffered formalin for 20 min and further permeabilised in 0.1% TritonX-100/PBS solution for 30 min. Sections were then incubated with primary rabbit anti–mouse ZO-1 antibody (1:100; ThermoFisher 61-7300) and rat anti-mouse CD32 antibody (EpCAM; 1:500; ThermoFisher 14-5791-81) overnight at 4 °C, followed by incubation with secondary Alexa Fluor 488 donkey anti-rat antibody (1:200; ThermoFisher A-21208) and Alexa Fluor 568 goat anti-rabbit antibody for 2 hrs at room temperature. To visualise cell nuclei, sections were incubated with DAPI (Sigma 10236276001) for 10 min according to the manufacturer’s instructions, and finally mounted using the Dako Fluorescence Mounting Medium (Agilent S302380-2). For the quantification of ZO-1 expression, representative images from immunofluorescent stained sections were taken on the Nikon C1 Invert confocal microscope (Nikon, Japan) at a magnification of 400x. Images were captured using a pattern which ensured unbiased selection and no overlap. Approximately 6-8 total images were captured for each colon section for analysis and averaged. ImageJ (NIH) was used for image processing to quantify the area of ZO-1 respective to area of DAPI staining.

*Flow cytometry of colon leukocytes*

Briefly, excised whole colon tissue was washed in phosphate buffered saline (PBS) to remove faecal matter, minced, further washed in Hank’s balanced salt solution (HBSS, Life Technologies), and digested with 0.5 mg/mL collagenase D for 60 min at 37 ^o^C. Digested tissues were passed through a 70 µm mesh and leukocytes enriched by resuspending cells in a 40% isotonic Percoll (Sigma), underlayed with 80% isotonic Percoll. Percoll gradients were resolved by centrifugation for 20 min at 1000 *g* without brake. Leukocytes were collected from the interface, washed with PBS and pelleted for staining.

**Supplementary Table 1. Centers of Disease Control (CDC) definitions of nosocomial infections**

| Infection | Criteria |
| --- | --- |
| Pneumonia   - Defined separately from other lower respiratory tract infections | Must meet one of the following criteria:   1. Physical examination findings AND change in sputum/organism isolated from blood cultures or sputum 2. Chest radio-graphical evidence AND change in sputum/organism isolated from blood cultures or sputum |
| Urinary tract infection | Must meet one of the following criteria:   1. One of the following: fever (>38°C), urgency, frequency, dysuria or suprapubic tenderness AND a urine culture of >10^5^ colonies/ml urine with no more than two species f organisms 2. Two of the following (>38°C), urgency, frequency, dysuria, or suprapubic tenderness AND dipstick positive for leukocytes or nitrites/pyuria/organisms seen on microscopy/two urine cultures with same uro-pathogen with >10^5^ colonies/ml/physician’s diagnosis |
| Lower respiratory tract infection (excluding pneumonia)  Includes:   - Bronchitis, tracheobronchitis, bronchiolitis, tracheltis - Lung abscess/empyema |  |

**Supplementary Table 2. Recorded pre-admission medications used by patients**

| Drug class | Examples |
| --- | --- |
| Antibiotics | All classes (including penicillins, tetracyclines, macrolides, fluoroquinolones, carbepenems) |
| Immunosuppressive medication:   - Glucocorticoids - Cytostatics:   - Alkylating agents  - Antimetabolites  - Cytotoxic antibodies   - Antibodies - Immunophilin-acting - Others | Prednisolone, budesonide  Cyclosporine  Methotrexate, azathioprine  Dactinomycin, bleomycin  Infliximab, rituximab, natalizumab  Tacrolimus, sirolimus  Mycophenolate, etanercept |
| Beta-blockers | Propranolol, bisoprolol |

**Supplementary Table 3. Primer sequences for the genes assessed using qRT-PCR**

| Gene | Forward sequence | Reverse sequence |
| --- | --- | --- |
| 18S | CTTAGAGGGACAAGTGGCG | ACGCTGAGCCAGTCAGTGTA |
| Occludin (Ocldn) | TTGAACTGTGGATTGGCAGC | CAAGATAAGCGAACCTTGGCG |
| Claudin 3 (Cldn3) | TACAAGACGAGACGGCCAAG | CTCAGACGTAGTCCTTGCGG |
| Claudin 5 (Cldn5) | GTGTCTGGTAGGATGGGTGG | GCGCCAGCACAGATTCATAC |
| junctional adhesion molecule-A (JAM-A) | CACCTCACTGTGCTTGTACCT | CACTTCGAGTACTGGGCTGG |
| Mucin 2 (MUC2) | CCATTGAGTTTGGGAACATGC | TTCGGCTCGGTGTTCAGAG |
| Mucin 4 (MUC4) | GCTCAAGTTGACAAGGAGCAGAGC | GGAGGACAAAAGAAGGCGTGGCC |
| Mucin 13 (MUC13) | GCCAGTCCTCCCACCACGGTA | CTGGGACCTGTGCTTCCACCG |

**Supplementary Figures**

**Supplementary Figure 1:** Bacterial load of **(A)** liver homogenates, **(B)** blood, **(C)** mesenteric lymph node (MLN) and **(D)** spleen from young and older mice were quantified 24 hrs after MCAO or sham-surgery (n=5-8/group). Data represent the mean ± SEM. Significance was determined by Mann–Whitney U-test and a p-value ≤ 0.05 was considered statistically significant.

**Supplementary Figure 2:** Gene expression of **(A)** occludin (*Ocldn*), **(B)** junctional adhesion molecule-A (*JAM-A*) and **(C)** claudin 3 (*Cldn3*) from the small intestine of young and older mice was analysed by qPCR 24 hrs after MCAO, and expressed as a fold change relative to that of young sham-operated controls (n>7/group). Data represent the mean ± SEM. Significance was determined by unpaired t-test. A p-value ≤ 0.05 was considered statistically significant.

**A**

**B**

**C**

**D**

**Supplementary Figure 3:** Gene expression of **(A)** claudin 3 (Cldn3), **(B)** claudin 5 (Cldn5) and **(C)** occludin (Occldn) from the colon of older mice was assessed 5 hrs after MCAO or sham surgery. Expression is represented as a fold change relative to that of sham-operated controls (n=7-8/group). In addition, the protein expression of **(D)** ZO-1 expression was assessed using immunofluorescent staining (n=4-5/group). Data represent the mean ± SEM. Significance was determined by unpaired t-test. A p-value ≤ 0.05 was considered statistically significant.

**Supplementary Figure 4:** Immune composition changes in the colon of young and older mice was assessed 5 hrs after MCAO or sham surgery. The number of cells per gram of colon tissue of key immune cell populations were quantified by flow cytometry at endpoint (n=5/group): **(A)** CD45^+^ cells, **(B)** CD3^+^ T-cells, **(C)** CD11b^+^ myeloid cells, **(D)** Ly6G^+^/Ly6C^-^ neutrophils and **(E)** Ly6G^-^/Ly6C^high^ monocytes. Data represent the mean ± SEM. Significance was determined by one-way ANOVA with post-hoc comparison and Holm-Sidak multiple testing correction. A p-value ≤ 0.05 was considered statistically significant: *p≤0.05, **p≤0.01.

**Supplementary Figure 5:** Protein expression of TNF-α and IL-10 in the **(A-B)** colon and **(C-D)** serum from young and older mice was quantified 24 hrs after MCAO or sham-surgery (n=4-7/group). Data represent the mean ± SEM. Significance was determined by unpaired t-test. A p-value ≤ 0.05 was considered statistically significant.

**Supplementary Figure 6:** A Streptomycin-resistant derivative of *E. coli* was orally inoculated into young and older mice 3 hrs after MCAO or sham surgery. The **(A)** duodenum, **(B)** jejunum and **(C)** ileum were assessed 24 hrs later for the presence and load of streptomycin-resistant *E. coli*. n=5-6/group. Data represent the mean ± SEM. Significance was determined by one-way ANOVA with post-hoc comparison and Holm-Sidak multiple testing correction. A p-value ≤ 0.05 was considered statistically significant.
